# Head circumference of children with sickle cell disease in Lagos, Nigeria

**DOI:** 10.11604/pamj.2016.25.4.8030

**Published:** 2016-09-13

**Authors:** Idowu Odunayo Senbanjo, Kazeem Adeola Oshikoya, Mohammed Salisu, Ijeoma Nnenna Diaku-Akinwumi

**Affiliations:** 1 Department of Paediatrics and Child Health, Lagos State University College of Medicine, PMB 21266, Ikeja, Lagos, Nigeria; 2 Department of Pharmacology, Lagos State University College of Medicine, Ikeja, Lagos, Nigeria

**Keywords:** Head circumference, sickle cell disease, growth standard

## Abstract

**Introduction:**

Growth retardation and under-nutrition are common in children with sickle cell disease (SCD). The aim of this study was to compare the head circumference (HC) of SCD children and non-SCD children and to determine the effect of malnutrition on head circumference of children with SCD.

**Methods:**

This was a prospective case-control study conducted at the Lagos State University Teaching Hospital, Nigeria, involving SCD children (subject, n = 118) and non-SCD children (control, n = 118) matched for age, sex, and socioeconomic status. Weight, height and HC were measured using standard techniques.

**Results:**

The mean ages of children with and without SCD were 7.46 ± 3.69 years and 7.01 ± 3.58 years, respectively. The HC increased significantly with age in both males and females (r = 0.75, p = < 0.001; r = 0.70, p < 0.001 respectively). There was no significant difference in HC between males and females (p > 0.05). At all ages, the mean head circumference of SCD children was not significantly (p > 0.05) different from non-SCD children. The In the age group 11-15 years, the prevalence of stunting was significantly higher among SCD children than non-SCD children. The mean HC of SCD children with stunting was significantly lower than those not stunted (51.7 vs. 53.5; P= 0.006) in age group 11.15 years.

**Conclusion:**

The head circumference of children with SCD is not significantly different from that of non-SCD children. Therefore, the HC chart for the general population is also applicable for monitoring head growth in children with SCD. The effect of malnutrition on head circumference of SCD children is most marked in age group 11-15 years.

## Introduction

Head circumference is a measurement of child’s head around its largest area [1]. It measures the distance from above the eyebrows and ears and around the back of the head [1]. It is useful for monitoring growth of the head because it is an early indicator of abnormalities of the brain and skull bone. The growth of the head is partly dependent on growth of the brain as well as growth of the skull bone. Thus, abnormalities of either the brain or the skull bone or both can affect head circumference. Therefore, measurement of head circumference is an important part of routine well baby care [2]. For clinical evaluation of head circumference, it is necessary to have population based reference standard with which to make comparison [3]. In addition, specific growth charts for head circumference are needed for certain clinical disorders such as Down syndrome, achondroplasia and sickle cell disease (SCD) which has as part of their manifestation impairment of growth of the head [4]. Sickle cell disease is an inherited disorder of haemoglobin [5]. It is the commonest genetic disorder in sub-Saharan Africa [5]. In many countries in Africa, 10-40% of the population carries the sickle-cell gene resulting in an estimated SCD prevalence of 2% [6]. In Nigeria, about one out every four persons are carriers and about 150,000 new cases of sickle cell disease are born every year making Nigeria the number one sickle cell endemic country in Africa [7, 8]. The disease affect virtually every system in the body through the process of chronic haemolysis, infarction and infection and this is usually associated with significant morbidity and mortality [5]. In patients with SCD, there is widening of the diploe and bossing of the skull bone as a result of chronic haemolytic anaemia which may culminate in the child having a big head [9]. On the other hand, there is the possibility of recurrent intracerebral sickling and thrombosis which may reduce brain growth and therefore the size of the head [9]. In addition, children with SCD are prone to under-nutrition due to increased metabolic demand [10]. This poor nutritional status may also influence growth of the brain. We hypothesize that the head circumference in children with SCD is different from that of apparently healthy children of the same age and gender. There is paucity of data comparing the head circumference of children with SCD and non-SCD children. Therefore, the aim of this study was to compare the head circumference values of children with SCD and apparently healthy children and to determine the effect of under-nutrition on head size of children with SCD.

## Methods

### Setting

This was a prospective case-control study carried out at the sickle cell disease clinic and general outpatient clinic of department of paediatrics and child health, Lagos State University Teaching Hospital (LASUTH), Ikeja, Lagos. The hospital is a tertiary health facility owned by the Lagos state government. It has about 200 bed space for children and is located in Ikeja Local Government Area. Health care need is provided free for children and the elderly. The hospital serves the inhabitants of Lagos state and the neighbouring Ogun state. The sickle cell disease clinic has a population of 120 patients. The clinic is usually run by four residents, two house officers, and one consultant. It is run every Thursday in a week and an average of one new patient is registered bi-weekly. Appointment for routine check-up is two to three months.

### Patient and selection

We selected consecutive patients with sickle cell disease who came to the clinic for routine follow up between March 5 and May 28, 2009. Patients were included in the study if the accompanying parents or guardians gave informed consent, in a stable state and do not require hospital admission. Each parent/guardian was interviewed in a private room by any of the researchers in accordance with a proforma specifically designed for the study. Information was obtained on demographic and socio-economic characteristics of the family. The families were classified into a socio-economic class according to the method of Ogunlesi et al [11]. The subjects were matched for age, sex and social class with controls of genotype AA recruited from the general outpatient clinic in a ratio of 1:1. The patients in the control group were those who came for routine follow-up clinic having being treated for illnesses such as malaria, upper respiratory tract infection (URTI), vernal conjunctivitis, otitis media, and urinary tract infection (UTI). Children with neurological disorders such as cerebral palsy and cerebro-vascular accidents were excluded. One hundred and eighteen patients with sickle cell disease and equal number of controls were recruited for the study. Of the sickle cell disease patients, 114 children had SS genotype and 4 had SC genotype.

### Ethical approval

Ethical clearance was obtained from the hospital research/ethics committee. While informed consent was obtained from the participants and parents or guardians. The ethical approval file number is LREC/10/06/297.

### Anthropometric measurement

The children were weighed using an electronic weighing scale calibrated in 100g units (SECA/UNICEF, Australia). Children who were too scared to stand on the scale were weighed together with the mother, and the mother’s weight automatically deducted to obtain the weight of the child. All children were weighed naked and to the nearest 0.1 kg. Length of children aged less than twenty-four months was measured using an infantiometer. This was done on a firm surface with assistance, usually by the mother. The knees were held down and the head held firmly against the headboard. These measurements were done to the nearest 0.1 cm. Height was measured using a height board for children aged twenty-four to fifty-nine months. This was done with the children standing erect without shoes, with eyes facing forward and the feet together on the horizontal plane. The head circumference was measured to the nearest 0.1cm with a non-stretchable tape using the glabella and the occiput as the landmarks. Standardization checks on the tools for anthropometric measurements were done periodically. Malnutrition in children was calculated from the degree of stunting (height-for-age) and thinness (BMI-for-Age) following World Health Organization (WHO) guidelines and cut off points [12]. In this study, a child was said to be stunted and thin if the Height-for-Age and BMI-for-Age were below minus two Standard Deviation (-2 SD) from the median of each international reference standard, respectively.

### Data analysis and presentation

Data was analysed by descriptive and inferential statistics using the SPSS for windows software version 11. The means and standard deviations (SD) were calculated for continuous variables while proportions were calculated for categorical variables. Independent Student t-test was used to determine differences in means between subjects and control. Categorical variables were compared using the Pearson Chi squared (χ^2^) test. A p-value of less than 0.05 or 95 % CI that does not include unity was accepted as statistically significant.

## Results

The mean ages of children with and without SCD were 7.46 ± 3.69 years and 7.01 ± 3.58 years, respectively. Sex distribution shows that 48.3% of SCD patients and 49.2% of non-SCD patients were male. SCD patients and non-SCD patients alike were mostly from lower socioeconomic class (84.7% vs 83.1%, respectively). There were no statistically significant differences in the mean ages, gender, and social classes of both the subjects and controls (p = 0.34, 0.90 and 0.72, respectively).

### Weight, height, BMI and nutritional status of SCD patients

Table 1 shows the means of the weight, height and BMI of children with SCD according to age groups and gender. In all the age groups, there was no statistically significant difference between genders for these parameters. The prevalence of stunting increase with age in both gender and this was statistically significant (χ^2^ = 12.0, p = 0.002) in females only while the prevalence of thinness increase significantly with age in both gender (p < 0.01). The prevalence of thinness was significantly higher in males than females among children in age group 6-10 years.

**Table 1:** Weight, height, body mass index and nutritional status of children with SCD*

	Age groups		
Parameters	≤ 5 years (n = 49)	6-10 years (n = 43)	11-15 years (n = 26)
**Weight (kg)**			
Males	14.0 (3.0)	23.0 (3.2)	29.1 (5.7)
Females	13.6 (3.1)	23.1 (3.6)	29.0 (7.1)
**Height (cm)**			
Males	97.8 (10.7)	127.3 (5.9)	138.8 (8.2)
Females	95.6 (11.7)	127.4 (8.8)	141.9 (12.6)
**BMI (kg/m^2^)**			
Males	14.5 (1.1)	14.2 (1.4)	15.0 (1.5)
Females	14.8 (1.0)	14.2 (0.9)	14.2 (1.3)
**Stunting**			
Male (%)	13.8	15.0	33.3
Female (%)	15.0	0.0	42.9
**Thinness**			
Male (%)	6.9	40.0†	50.0*
Female (%)	10.0	13.0	64.3*

* Values are mean (SD) unless otherwise stated; SCD = sickle cell disease

* p < 0.05 for age group difference

† p < 0.05 for gender difference

### Comparing nutritional status of SCD patients and apparently healthy children

Table 2 compares the nutritional status of SCD and non-SCD children. In age group 11-15 years, the prevalence of stunting (38.5% vs. 0.0%, p=0.002) and thinning (57.7 % vs. 15.8%, p=0.005) were significantly higher among SCD children than in non-SCD children.

**Table 2:** Comparing nutritional status of SCD and non-SCD children according to age groups and gender

Parameters	Age groups					
	< 5 years		6-10 years		11-15 years	
	SCD (n=49)	non-SCD (n=52)	SCD (n=43)	non-SCD (n=47)	SCD (n=26)	non-SCD (n=19)
**Stunted**	7 (14.3)	3 (5.8)	2 (4.7)	2 (4.3)	10 (38.5)	0 (0.0)
**Not Stunted**	42 (85.7)	49 (94.2)	41 (95.3)	45 (95.7)	16 (61.5)	19 (100.0)
**Thin**	4 (8.2)	5 (9.2)	11 (25.6)	7 (14.9)	15 (57.7)	3 (15.8)
**Not Thin**	45 (91.8)	47 (90.8)	32 (74.4)	40 (86.1)	11 (42.3)	16 (84.2)

SCD = sickle cell disease

### Head circumference of SCD patients

Table 3 compares the mean HC according to age groups and gender. There was no significant difference in HC between males and females in all the age groups. The HC increased significantly with age in both males and females (r = 0.75, p = < 0.001; r = 0.70, p < 0.001 respectively). There was also a significant positive correlation of HC with height and weight in both genders (r = 0.78, p = < 0.001; r = 0.78, p < 0.001 respectively). Table 4 compares the mean HC of children with SCD and apparently healthy children according to age in males and females respectively. At every age, there was no significant difference in HC between children with SCD and apparently healthy children in both genders.

**Table 3:** Head circumference values of children with sickle cell disease

Age groups (Years)	Male			Female			p-value
	No	Mean HC ±SD (cm)	95% CI	No	Mean HC ± SD (cm)	95% CI	
**< 5**	29	49.6 ± 1.6	49.0 - 50.2	20	48.8 ± 1.6	48.1 - 49.6	0.112
**6-10**	20	52.6 ± 1.4	51.9 - 53.2	23	52.0 ± 1.6	51.3 - 52.6	0.185
**11-15**	12	53.4 ± 1.6	52.4 - 54.4	14	52.3 ± 1.7	51.3 - 53.3	0.102

HC = Head circumference; SD = standard deviation; CI = confidence interval

**Table 4:** Comparing the head circumference values of children with SCD and apparently healthy children by age and gender

Gender	Age (year)	Sickle cell disease subjects		Non-sickle cell disease subjects		
		No	Head circumference	No	Head circumference	P-value
**Male**	1	4	47.6	3	48.0	0.830
	2	5	48.3	10	48.7	0.562
	3	7	50.4	6	49.8	0.585
	4	8	49.9	4	50.3	0.691
	5	5	50.8	3	51.5	0.460
	6	1	53.0	2	52.3	0.667
	7	6	53.2	2	50.8	0.283
	8	2	52.3	6	51.5	0.388
	9	3	52.7	6	51.9	0.516
	10	8	52.1	9	52.9	0.392
	11	7	53.4	8	52.0	0.092
	12	3	53.4	-	-	-
	13	2	53.5	1	53.0	0.879
**Female**	1	4	47.4	2	46.5	0.667
	2	2	47.5	9	48.6	0.240
	3	6	48.6	5	48.2	0.700
	4	4	50.1	6	49.8	0.333
	5	4	50.0	4	49.8	0.809
	6	2	51.5	3	50.8	0.739
	7	7	51.9	-	-	-
	8	5	52.2	7	51.1	0.403
	9	2	52.5	4	53.4	0.669
	10	7	51.9	8	52.7	0.189
	11	5	52.3	6	51.7	0.589
	12	6	51.7	4	54.1	0.063
	13	3	53.7	-	-	-

SCD = sickle cell disease

### Effect of Nutritional status on head circumference

Table 5 shows the mean HC values of children with SCD according to their nutritional status and age groups. In age group 11-15 years, the mean HC was significantly lower (51.7 vs. 53.5; P= 0.006) among those children with stunting than those not stunted.

**Table 5:** Mean (SD) of HC in SCD patients according to nutritional status and age groups

Variables	≤ 5 years (n = 49)	6-10 years (n = 43)	11-15 years (n = 26)
**Height-for-Age z-score**			
Stunted	48.5 (1.6)	52.3 (1.0)	51.7 (1.4)
Not stunted	49.4 (1.7)	52.2 (1.5)	53.5 (1.5)
P-value	0.192	0.885	0.006
**BMI-for-Age z-score**			
Thin	48.6 (1.6)	51.7 (1.4)	52.4 (1.7)
Not thin	49.3(1.7)	52.4 (1.5)	53.4 (1.6)
P-value	0.448	0.126	0.144

SCD = sickle cell disease ; SD = standard deviation

## Discussion

The HC is one of the most common anthropometric measurements routinely carried out at the children welfare clinic for assessment and monitoring of brain growth and development. It is simple, highly reliable and re-producible [13]. Similar to the findings in non-SCD children, the HC of children with SCD increased with age in both genders [14, 15]. These indicate normal brain growth and maturation. In apparently healthy children, the growth velocity of head circumference is highest in the first 3 years of life [15]. Our study, though not longitudinal, shows that head growth was highest between ages of 1 and 6 years with an increment of 5.4cm in boys and 4.1cm in girls. This findings is similar to the findings in our control subjects. The head circumference of males in this study was larger than that of the females. This is also similar to the findings in other studies [14, 15] and this difference has been ascribed to the general theory of influence of the Y-chromosome on body growth [14]. There are few studies that compares the HC of SCD patients with HC for apparently healthy children and their findings are conflicting [16–18]. Our study shows that the HC of children with SCD was not significantly different from the HC for apparently healthy children of similar age and gender. This is similar to findings in previous study carried in a different health facility in Lagos, Nigeria [16]. However, in similar studies in Kenya [17] and Jamaica [18], the mean head circumference was higher among children with sickle cell disease with children from lower socio-economic class having higher mean values. The degree of bossing of skull bone in children with sickle cell disease is related to the rate of haemolysis and increased haematopoiesis. The reason for the different findings in these studies therefore might be related to the level of care received by these children. This is instructive because it shows that the development of specific HC chart for children with SCD should take into consideration these factors.

The nutritional status of children with SCD has been extensively studied particularly in North America, Caribbean and Europe [10]. Most of these studies shows evidence of growth failure and nutrients´ deficiencies in children with SCD [10]. In our study, the magnitude of under-nutrition in SCD patients is high but was similar to that seen in non-SCD patient during preschool age and mid-childhood while in adolescents, the SCD patients have higher prevalence of stunting and thinning than non-SCD. This is similar to the findings in other studies [16, 19]. The reason adduced for this is that the adolescents have had the disease for a longer period and the sequel of the disease is likely to be more pronounced in them. However, other factors such as the influence of quantity and quality of dietary intake during adolescence period compared with preschool and mid-childhood may need to be investigated to determine their contributions to the high prevalence of under-nutrition in this age group. The nutritional status of a child has influence on head growth. Nutrient deficiency can alter electrophysiological function of the brain thereby affecting the memory, cognitive function, academic achievement and intellectual ability of a child [20]. In this study, the mean HC values of children with SCD who were stunted was smaller than those not stunted. This effect on HC was more pronounced in the age group where malnutrition has its toll on SCD patients. This is similar to the findings in non-SCD patients by Oyedeji et al [21] where the mean head circumference values of malnourished children was significantly lower than well nourished children. Various studies have documented negative impact of SCD on academic achievement of affected children [22, 23]. This is believed to probably be due to the direct effect of SCD on intellectual ability of the child through cumulative effect of recurrent subclinical, intracerebral sickling and thrombosis of the brain as well as through other mechanisms that are yet to be identified. In a study by Eriksen HF et al [24], the HC was proven to be a significant predictor of child intelligent quotient. Therefore, in further studies to unravel the cause of poor academic performance in SCD patients, It will be important to link the effect of growth failure and nutritional deficiencies on intellectual capability in SCD patients. The limitation of this study is the small sample size of SCD patients. A larger sample may be needed to further support the findings in this study.

## Conclusion

The HC of children with SCD is not significantly different from HC of non-SCD children. This indicates that the HC for general paediatric population may be useful for assessment of nutritional status and detecting developmental anomalies in children with SCD In addition, our study revealed that the prevalence of under-nutrition is higher among children with SCD than non-SCD children especially during the adolescent period. During this period, there is a marked effect of chronic malnutrition on the head size of children with SCD. There is a need for further research studies on the effect of growth failure and nutritional deficiencies on the head size, intellectual capability and academic performance of children with SCD.

### What is known about this topic

- Growth deficiency is common among children with SCD;
- Monitoring growth of the head is an early indicator of abnormalities of the brain and skull bone;
- Conflicting reports on normative value of HC in children with SCD.

### What this study adds

- HC of children with SCD was similar to that of apparently healthy children;
- Stunted SCD children had significantly reduced HC.

## References

[1] Kimmel SR, Ratliff-Schaub K, Rakel RE (2007). Growth and development. Textbook of Family Medicine..

[2] Zahl SM, Wester K (2008). Routine measurement of head circumference as a tool for detecting intracranial expansion in infants: what is the gain? A nationwide survey. Pediatrics..

[3] de Onis M, Onyango AW, Borghi E, Garza C, Yang H, WHO Multicentre Growth Reference Study Group (2006). Comparison of the World Health Organization (WHO) Child Growth Standards and the National Center for Health Statistics/WHO international growth reference: implications for child health programmes. Public Health Nutr..

[4] Ranke MB (1989). Disease-specific growth charts--do we need them?. Acta Paediatr Scand Suppl..

[5] Diallo DA, Guindo A (2014). Sickle cell disease in sub-Saharan Africa: stakes and strategies for control of the disease. Curr Opin Hematol..

[6] Modell B, Darlison M (2008). Global epidemiology of haemoglobin disorders and derived service indicators. Bulletin of the WHO..

[7] World Health Organization (2006). Report by the Secretariat of the Fifty-ninth World Health Assembly A59/9.

[8] Roberts I, de Montalembert M (2007). Sickle cell disease as a paradigm of immigration hematology: new challenges for hematologists in Europe. Haematologica..

[9] Rodgers GP (1997). Overview of pathophysiology and rationale for treatment of sickle cell anemia. Semin Hematol..

[10] Al-Saqladi AW, Cipolotti R, Fijnvandraat K, Brabin BJ (2008). Growth and nutritional status of children with homozygous sickle cell disease. Ann Trop Paediatr..

[11] Ogunlesi TA, Dedeke IOF, Kuponiyi OT (2008). Socio-economic classification of children attending specialist paediatric centres in Ogun state, Nigeria. Niger Med Pract..

[12] World Health Organization expert committee (1995). Physical status, the use and interpretation of anthropometry. WHO technical report series..

[13] Sullivan JC, Tavassoli T, Armstrong K, Baron-Cohen S, Humphrey A (2014). Reliability of self, parental, and researcher measurements of head circumference. Mol Autism..

[14] Zaki ME, Hassan NE, El-Masry SA (2008). Head circumference reference data for Egyptian children and adolescents. East Mediterr Health J..

[15] Elmali F, Altunay C, Mazicioglu MM, Kondolot M, Ozturk A, Kurtoglu S (2012). Head circumference growth reference charts for Turkish children aged 0-84 months. Pediatr Neurol..

[16] Oredugba FA, Savage KO (2002). Anthropometric finding in Nigerian children with sickle cell disease. Pediatr Dent..

[17] Mpaata PJ (1983). An Anthropometric Study Of Children With Sickle Cell Anaemia At The Kenyatta National Hospital..

[18] Clarke JM (1977). Homozygous sickle cell disease in Jamaica in the first year of life..

[19] Al-Saqladi AW, Bin-Gadeen HA, Brabin BJ (2010). Growth in children and adolescents with sickle cell disease in Yemen. Ann Trop Paediatr..

[20] Ivanovic DM, Leiva BP, Pérez HT, Olivares MG, Díaz NS, Urrutia MS, Almagià AF, Toro TD, Miller PT, Bosch EO, Larraín CG (2004). Head size and intelligence, learning, nutritional status and brain development, Head, IQ, learning, nutrition and brain. Neuropsychologia..

[21] Oyedeji GA, Olamijulo SK, Osinaike AI, Esimai VC, Odunusi EO, Aladekomo TA (1997). Head circumference of rural Nigerian children--the effect of malnutrition on brain growth. Cent Afr J Med..

[22] Epping AS, Myrvik MP, Newby RF, Panepinto JA, Brandow AM, Scott JP (2013). Academic attainment findings in children with sickle cell disease. J Sch Health..

[23] Ogunfowora OB, Olanrewaju DM, Akenzua GI (2005). A comparative study of academic achievement of children with sickle cell anemia and their healthy siblings. J Natl Med Assoc..

[24] Eriksen HL, Kesmodel US, Underbjerg M, Kilburn TR, Bertrand J, Mortensen EL (2013). Predictors of intelligence at the age of 5: family, pregnancy and birth characteristics, postnatal influences, and postnatal growth. PLoS One..

